# Mechanoregulatory role of TRPV4 in prenatal skeletal development

**DOI:** 10.1126/sciadv.ade2155

**Published:** 2023-01-25

**Authors:** Nidal S. Khatib, James Monsen, Saima Ahmed, Yuming Huang, David A. Hoey, Niamh C. Nowlan

**Affiliations:** ^1^ Department of Bioengineering, Imperial College London, London, UK.; ^2^ Department of Mechanical, Manufacturing, and Biomedical Engineering, School of Engineering, Trinity College Dublin, Dublin, Ireland.; ^3^ Trinity Centre for Biomedical Engineering, Trinity Biomedical Sciences Institute, Trinity College Dublin, Dublin, Ireland.; ^4^ Advanced Materials and Bioengineering Research Centre (AMBER), Royal College of Surgeons in Ireland and Trinity College Dublin, Dublin, Ireland.; ^5^ School of Mechanical and Materials Engineering, University College Dublin, Dublin, Ireland.; ^6^ UCD Conway Institute, University College Dublin, Dublin, Ireland.

## Abstract

Biophysical cues are essential for guiding skeletal development, but the mechanisms underlying the mechanical regulation of cartilage and bone formation are unknown. TRPV4 is a mechanically sensitive ion channel involved in cartilage and bone cell mechanosensing, mutations of which lead to skeletal developmental pathologies. We tested the hypothesis that loading-driven prenatal skeletal development is dependent on TRPV4 activity. We first establish that mechanically stimulating mouse embryo hindlimbs cultured ex vivo stimulates knee cartilage growth, morphogenesis, and expression of TRPV4, which localizes to areas of high biophysical stimuli. We then demonstrate that loading-driven joint cartilage growth and shape are dependent on TRPV4 activity, mediated via control of cell proliferation and matrix biosynthesis, indicating a mechanism by which mechanical loading could direct growth and morphogenesis during joint formation. We conclude that mechanoregulatory pathways initiated by TRPV4 guide skeletal development; therefore, TRPV4 is a valuable target for the development of skeletal regenerative and repair strategies.

## Teaser

Biophysical regulation of joint cartilage growth and shape during skeletal development depends on TRPV4 activity.

## INTRODUCTION

Skeletogenesis begins in early embryonic development when the entire skeleton forms first as a cartilaginous template (*1*, *2*). The cartilage anlagen are gradually mineralized over prenatal and postnatal development to form the mature skeleton (*3*). The ultimate size, shape, and organization of skeletal tissues depends on many cellular processes, including matrix production, cell division, hypertrophy, intercalation, and changes in cell size and orientation (*4*–*8*). The spatial and temporal orchestration of such a complex process depends on an interplay between genetic and epigenetic mechanisms influenced by environmental factors, notably mechanical signals (*9*, *10*). Skeletogenesis in later development is influenced by fetal movements such as kicking, which induce stresses and strains on the developing skeleton (*11*). Reduced or absent fetal movement is associated with skeletal abnormalities such as hip dysplasia, joint contractures (fixed joints), and slender, hypo-mineralized bones (*12*–*14*). Animal models of fetal immobilization or reduced muscle display skeletal abnormalities akin to that found in akinesia syndromes, including shorter limbs with reduced bone, failed joint cavitation and joint shape malformation (*6*, *9*, *15*–*19*). While substantial progress has been made mapping the genetic molecular control of skeletal development (*20*, *21*), we still have a poor understanding of the mechanisms underlying the influence of mechanical factors. Identifying key mechanoregulatory mechanisms of skeletogenesis would be valuable for advancing our understanding of biophysical developmental control and motivate developmentally inspired mechanotherapeutic or skeletal tissue regeneration strategies.

Skeletal cells such as chondrocytes and bone cells sense and respond to mechanical stimuli as a means of regulating skeletal tissue growth, homeostasis, and repair (*22*). Mechanical loading of skeletal tissues exposes cells to multiple modes of stress, such as compression, tension, hydrostatic pressure, osmotic pressure, and fluid shear. In a process called mechanotransduction, biophysical cues are detected by specialized mechanosensors that convert them into biochemical signals, consequently regulating cell behavior (*23*–*25*). Cell membrane mechanosensors detect physical changes at the cell membrane, such as integrins, which sense cell-matrix interactions through focal adhesions with the substrate (*26*), cadherins, which transduce cell-cell interactions (*27*), and mechanosensitive ion channels, which activate in response to physical changes in the membrane such as tension (*28*), hypotonicity (*29*), deflection of cell-matrix focal adhesions (*30*), and deflection of the primary cilia (*31*). While integrins, cadherins, and ion channels have been shown to regulate homeostatic cell activities, such as proliferation, survival, maturation, and matrix deposition in skeletal cells (*32*–*34*), their involvement in mechanically regulated processes of cartilage and bone development remains unclear.

The involvement of mechanosensitive ion channels in skeletal development has been highlighted by the effects of hereditary functional mutations (channelopathies) of transient receptor potential vanilloid 4 (TRPV4). TRPV4 is a polymodal Ca2^+^-permeable nonselective ion channel (*35*), mutations in which leads to a phenotypically diverse range of severe skeletal conditions, including lethal metatropic dysplasia, spondylometaphyseal dysplasia (dwarfism), and autosomal dominant brachyolmia (*36*–*38*). First discovered in 2000 (*39*, *40*), TRPV4 was initially found to be responsible for transducing osmotic signals (*29*), but has since been implicated in the cell biosynthetic response to compressive loading (*41*), hydrostatic pressure (*42*), and oscillatory fluid shear (*31*). Previous work has found a critical role for TRPV4 in cartilage and bone cell function in vitro. Chemical activation of the ion channel using specific channel agonists stimulates up-regulation of chondrogenic gene expression markers (*ACAN* and *COL2*α*1*) and the production of cartilaginous matrix proteins glycosaminoglycans and collagen (*31*, *41*, *43*). Up-regulated TRPV4 activity has also been associated with chondrogenic differentiation of mesenchymal stem cells (MSCs), induced pluripotent stem cells (iPSCs), and iPSC-derived chondroprogenitors (*41*, *43*–*45*), indicating a role of TRPV4 in chondrogenesis. *Trpv4*^−/−^ knockout mice develop spontaneous osteoarthritis at a younger age than wild-type controls (*46*), while cartilage-specific Trpv4^−/−^ mice experience reduced severity of aging-associated osteoarthritis than wild-type controls (*47*). In bone, TRPV4-mediated signaling has been shown to regulate osteocyte differentiation (*48*), while *Trpv4*^−/−^ knockout mice exhibit reduced bone loss and reduced osteoclast function in an unloading-induced bone loss model (*49*), suggesting a key involvement of TRPV4 in regulating bone adaptation to loading. While previous work has revealed an involvement of TRPV4 in both skeletal developmental diseases and mature skeletal cell activity in vitro, the specific function of the ion channel has not yet been characterized in developing skeletal tissues.

Major advances in TRPV4 agonist and antagonist discovery in recent years and successful evaluation of the first selective TRPV4 antagonist in clinical trials (*50*, *51*) provide a promising view of the development of human therapies. Furthermore, TRPV4 has been recognized as a potentially valuable therapeutic target for joint diseases (*52*, *53*); therefore, a better understanding of how TRPV4 functions in skeletal tissue development may be critical for informing therapies targeting TRPV4 mutation–associated disease or skeletal regeneration in the near future. In this study, we test the hypothesis that mechanically regulated cartilage formation and mineralization of developing hindlimb skeletal tissues are mediated by TRPV4 mechanotransduction. We first quantify the effects of mechanical loading on cartilage growth, morphogenesis, and diaphyseal mineralization of embryonic murine hindlimb explants cultured ex vivo using a mechanostimulation bioreactor system. Next, we determine whether mechanoregulation of skeletal development is dependent on TRPV4 activity. Last, we elucidate the involvement of TRPV4 in the physiological link between mechanical loading and cartilage growth, cell proliferation, and matrix biosynthesis during skeletal development.

## RESULTS

### Dynamic stimulation of mouse embryo limb explants promotes cartilage growth and morphogenesis but not diaphyseal mineralization

**Fig. 1.**
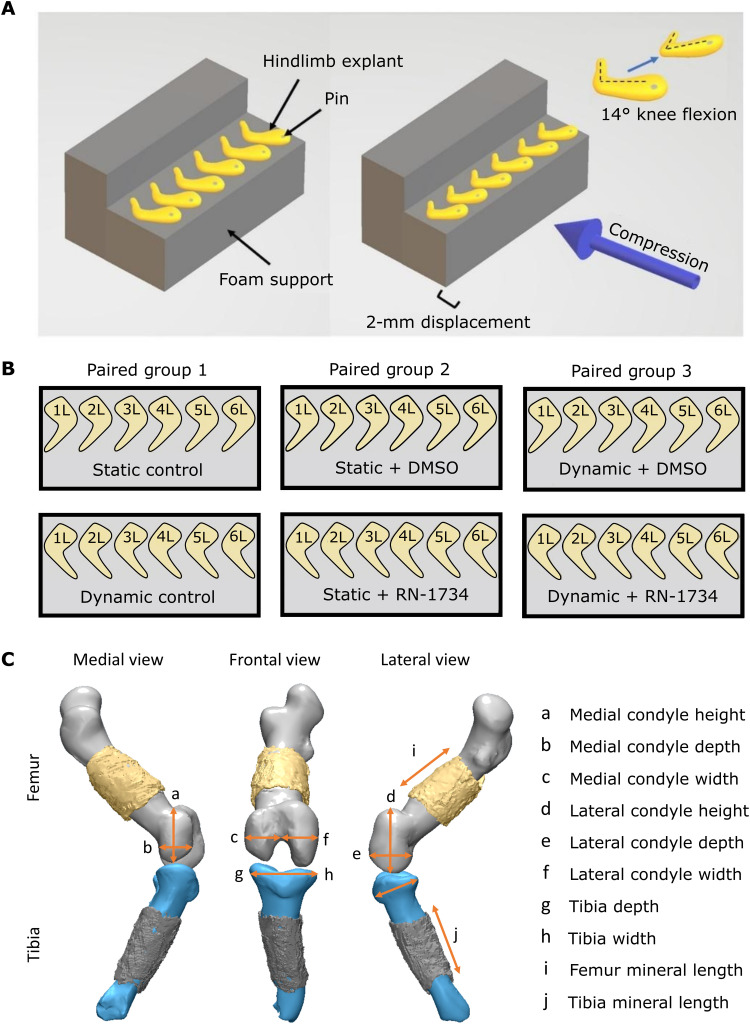
Experimental setup for the mechanical stimulation of mouse hindlimbs, comparison groups, and visualization of measurements for quantitative analysis. (**A**) Six to eight limbs pinned to foam supports were placed within bioreactor chambers for dynamic culture or petri dishes for static culture. Dynamic cultured limbs were exposed to cyclic flexion-extension movements of approximately 14° (±2°) at 0.67 Hz (*54*), applied by compressive displacement of the foam supports (movie S1). (**B**) Within each comparison group for the cartilage growth and mineralization experiments, contralateral limbs from the same embryo served as paired samples for three comparison groups. (**C**) Eight cartilage joint features and two mineral length measurements were measured from 3D cartilage and mineral models generated from OPT data.

The first step of this research established the effects of dynamic mechanical loading on cartilage growth, shape, and mineralization of hindlimb explants from mouse embryos. We previously developed a bioreactor system to apply dynamic mechanical loading regimes to embryonic chick limb explants ex vivo, establishing a positive effect of loading on joint morphogenesis (*54*, *55*) and diaphyseal mineralization (*55*). This model permits rigorous control over the mechanical stimuli applied to developing skeletal tissues, a major challenge in in ovo or in utero animal model studies (*56*). In this study, we used mouse embryo hindlimbs in our mechanostimulation bioreactor system, which permits investigation of the distinct mammalian processes of bone formation from avian development and opens up avenues for culturing limbs from transgenic mice. To determine the effect of dynamic culture on skeletal development ex vivo, embryonic day 15.5 mouse hindlimbs were cultured for 6 days, with one limb in dynamic culture conditions and the contralateral limb in static conditions (Fig. 1, A and B, and movie S1). Limbs were stained with Alcian blue (cartilage) or Alizarin red (mineral), imaged in three-dimensional (3D) using an optical projection tomography (OPT) approach (*57*), and then segmented to generate cartilage and mineral 3D models. Rudiment length and joint shape parameters were measured from the models (Fig. 1C).

**Fig. 2.**
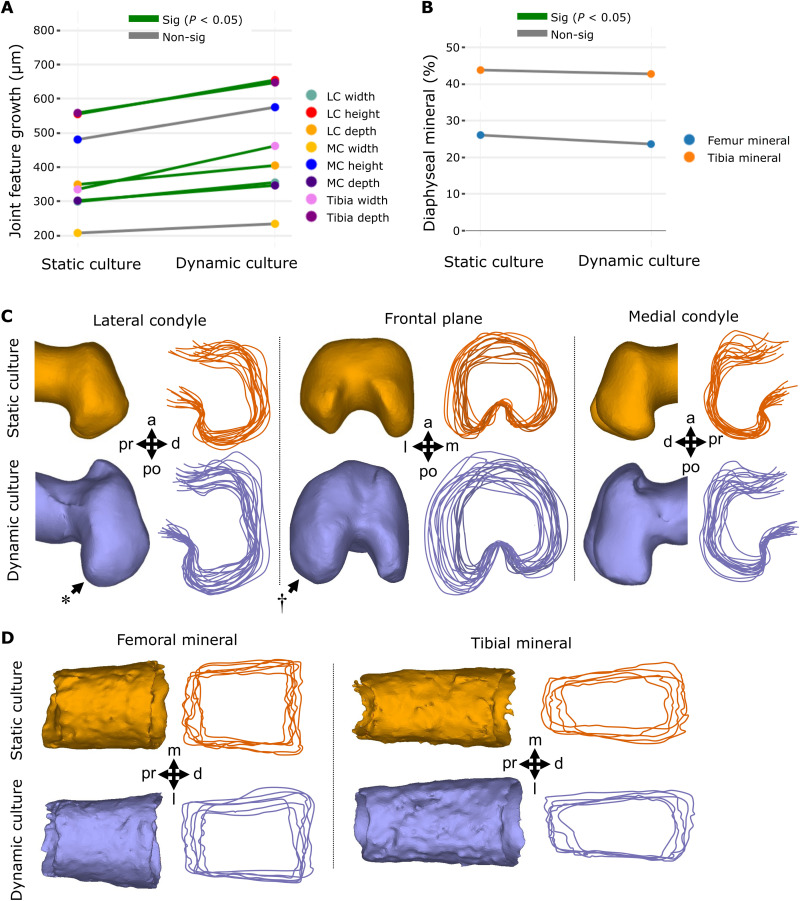
Dynamic loading of cultured mouse embryo hindlimbs promotes joint cartilage feature growth and shape but not the extent of mineralization or bone collar shape. (**A**) Average paired differences in growth for joint cartilage features (each line represents one of eight features) in static versus dynamic culture (*n* = 8 limbs per group). Green lines, significant (*P* < 0.05) feature differences; gray lines, nonsignificant feature differences. (**B**) Average extents of femur (*n* = 6) and tibia mineralization (*n* = 6) in static and dynamic cultured paired limbs. (**C**) Representative samples and joint shape contours for distal femora cultured in static and dynamic conditions. Arrows indicate regions of increased growth and shape development. * represents more prominent posterior curl observed in dynamically loaded limbs. † represents larger lateral expansion found in the lateral condyle of dynamically loaded limbs. (**D**) Representative samples and outlines of femoral and tibial bone collars from limbs cultured in static and dynamic conditions. Pr, proximal; d, distal; a, anterior; po, posterior; m, medial; l, lateral; MC, medial condyle; LC, lateral condyle.

Mechanical loading induced by dynamic culture significantly increased growth of six of eight knee joint shape features compared to static controls (*P* < 0.05, *n* = 8), namely, the width, height, and depth of the lateral condyle, tibial depth and width, and medial condyle depth (Fig. 2A and fig. S1.1). Medial condyle height and width were not significantly increased by dynamic loading, but trends of increased growth of these features with loading were evident. Visual analysis of the shape outlines revealed that the most prominent effects of dynamic culture on joint shape were the increased definition of the posterior curl of the lateral condyle (Fig. 2C, *) and mediolateral expansion of the lateral condyle (Fig. 2C, †). From these data, we conclude that growth and morphogenesis of the joint cartilage of cultured explants are influenced by mechanical loading in our culture system.

In contrast to reported increases in the diaphyseal mineralization of chick limb explants in response to mechanical stimuli (*55*, *58*), dynamic loading of cultured murine limbs had no significant effects on diaphyseal femoral or tibial mineral length (Fig. 2B and fig. S2). Furthermore, no visible differences were observed in bone collar shape between dynamically and statically cultured mouse femora or tibiae (Fig. 2D). Together, our findings indicate that dynamic loading of ex vivo mouse embryo limbs stimulates cartilage growth and morphogenesis but not mineralization. The differences found in the mineralization response to loading across the mouse and chick model are likely due to the reliance of endochondral ossification on blood vessel invasion in the mammalian limb (*3*, *59*). In contrast to mammalian osteogenesis, avian long bones do not require vascularization of the primary cartilage before mineralization (*59*). Since the vasculature in our hindlimb explants becomes nonfunctional after dissection, it is possible that osteogenic progenitors do not reach the ossification sites in our murine cultured hindlimbs. In summary, we have validated a powerful model of ex vivo mouse limb development, which captures key stages of mechanically driven cartilage growth and joint morphogenesis.

### TRPV4 expression is mechanically regulated in developing murine cartilage

TRPV4 expression has been identified in most adult skeletal cell types (*48*), but the variance and patterning of its expression in developing skeletal tissues have not previously been investigated. To characterize the spatial expression of TRPV4 in prenatal skeletal tissues and determine how it is affected by ex vivo loading, we compared the localization of TRPV4 protein concentrations between statically and dynamically cultured hindlimb explants. Cultured femora were sectioned and labeled for TRPV4 at the protein level using immunofluorescence staining. TRPV4 was found to be colocalized to the cell membrane in murine chondrocytes (fig. S3). Three regions of the femur were imaged and quantitatively analyzed, namely, the medial condyle, lateral condyle, and growth plate. Individual cell TRPV4 intensities were measured across all chondrocytes captured within cropped areas of these three regions.

**Fig. 3.**
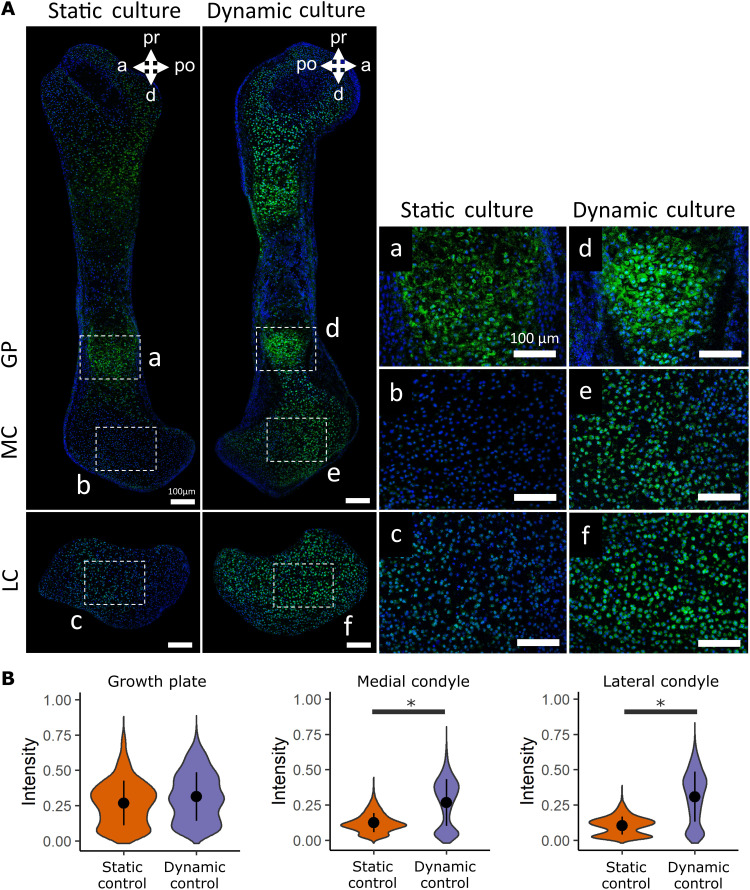
TRPV4 protein expression is mechanically regulated in developing joint cartilage. (**A**) Immunofluorescence staining of the TRPV4 protein in the growth plate (GP; a and d), medial condyles (b and e), and lateral condyles (c and f) of femora subjected to static and dynamic culture conditions (*n* = 3 limbs per group). Blue, 4′,6-diamidino-2-phenylindole (DAPI); green, TRPV4. Scale bars, 100 μm. (**B**) Quantification of pooled cell TRPV4 protein immunofluorescence intensity within the growth plate, medial condyle, and lateral condyle. * indicates a significant difference between groups (*P* < 0.05).

In statically cultured limbs, TRPV4 expression was highly expressed in the hypertrophic cartilage of the growth plate but appeared less prominent in metaphyseal and joint cartilage (Fig. 3A). Dynamic loading led to a marked increase in the prominence of TRPV4 expression in the medial and lateral condyles compared to limbs cultured in static conditions, while TRPV4 expression in the growth plate was not affected by loading when comparing across all samples (Fig. 3A and fig. S4). TRPV4 expression was spatially varied throughout the dynamically loaded condyles, with increased expression in the anterior aspect of the medial condyle and in the anterior and proximal aspects of the lateral condyle. Quantitative data on TRPV4 intensity revealed a significant increase in intensity for the medial and lateral condyles due to dynamic loading but no change in intensity at the growth plate (Fig. 3B). These data demonstrate that in ex vivo–cultured embryonic limb explants, TRPV4 protein concentration is mechanically up-regulated in joint epiphyseal chondrocytes, while regulation of TRPV4 in hypertrophic chondrocytes is independent of extrinsic loading.

**Fig. 4.**
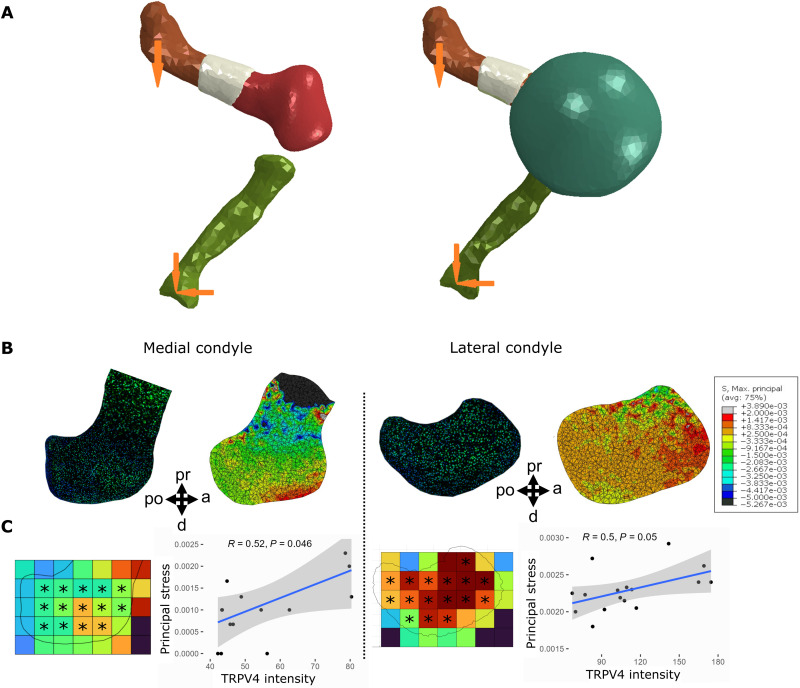
High TRPV4 expression is localized to regions of high maximum principal stress. (**A**) FE model setup without the joint capsule (left) and with the capsule (right). Maximum principal stresses were calculated through FE analysis of an ideal limb (ABAQUS), generated from the average 3D shape of multiple registered 3D models. Models of the distal femur, bone collar, and proximal tibia were oriented to match the angle of limbs in the zero position at day 6 of culture. (**B**) Predicted spatial patterns of maximum principal stress in the medial (left) and lateral (right) condyles from FE models of dynamically cultured mouse hindlimb distal femora with equivalently oriented sample sections of most similar shape stained for TRPV4. (**C**) TRPV4 expression intensity correlated with maximum principal stresses in the medial and lateral condyles (*R* = 0.5/0.52, *P* ≤ 0.05). TRPV4 expression intensity (for the closest matching sample shape to the ideal limb) and maximum principal stresses across the medial condyle (left) and lateral condyle (right) were averaged into 100-μm bins. Average values from regions of interest (*) were correlated and evaluated using Pearson’s correlation coefficient (*R*) with 95% confidence bands.

To further interrogate the association between biophysical stimuli and TRPV4 expression, we examined whether TRPV4 preferentially localized to sites within the femoral condyles that experience high stress during loading. A 3D finite element (FE) model of the dynamically loaded limb explants was constructed to compute the maximum principal stress distributions within the distal femur (Fig. 4, A and B). The relationship between TRPV4 protein expression and stress was quantitatively assessed by correlating TRPV4 intensity and stress values averaged into 100-μm grid locations spanning the femoral condyles (Fig. 4C). Maximum principal stresses were highest in the anterior aspect of the lateral condyle and lowest in the hypertrophic region adjacent to the mineralized cartilage (Fig. 4B). Stresses were generally higher in the lateral condyle than in the medial condyle, potentially correlating with increased mechanically mediated growth of the lateral condyle than the medial (Fig. 2). Regions of high stress in the femoral condyles colocalized with regions of increased TRPV4 intensity (Fig. 4B). Maximum principal stresses correlated with TRPV4 expression intensity in both the medial (*R* = 0.52 and *P* < 0.05) and lateral (*R* = 0.5 and *P* = 0.05) condyles. Stresses were orders of magnitude higher at the anterior-distal aspect of the medial condyle compared to the rest of the condyle, which corresponded to higher TRPV4 expression in the anterior region relative to the rest of the condyle (Fig. 4B). In the lateral condyle, a pronounced reduction in maximum principal stress in the distal prominence of the lateral condyle relative to the rest of the condyle was associated with reduced TRPV4 expression intensity in this region compared to the rest of the condyle (Fig. 4B). Colocalization between high maximum principal stresses and elevated TRPV4 expression was not observed in the hypertrophic region, as although stresses were very low in the hypertrophic region due to stress shielding from the mineralized cartilage (Fig. 4B), TRPV4 expression was very high in the hypertrophic chondrocytes. The lack of difference in TRPV4 expression between dynamically and statically cultured growth plates indicates that TRPV4 in hypertrophic chondrocytes may be regulated by nonmechanical pathways or by cell- or tissue-intrinsic mechanical signals. Together, these findings indicate that the spatial variation of biophysical stimuli generated by dynamic loading ex vivo drives local concentrations of TRPV4 in the cartilaginous epiphysis, but not at the growth plate.

### Joint cartilage growth and shape are mediated by TRPV4 activity

Having shown that murine joint cartilage growth and TRPV4 protein expression are mechanically regulated, we next set out to determine whether loading-induced joint cartilage growth is dependent on TRPV4 activity. A TRPV4-specific antagonist, RN-1734, was used to block TRPV4-mediated Ca^2+^ signaling (*60*, *61*) in statically and dynamically cultured limb explants. One hindlimb from each animal was cultured with 10 μM RN-1734, while the contralateral limb was cultured with the drug vehicle [dimethyl sulfoxide (DMSO)] alone.

**Fig. 5.**
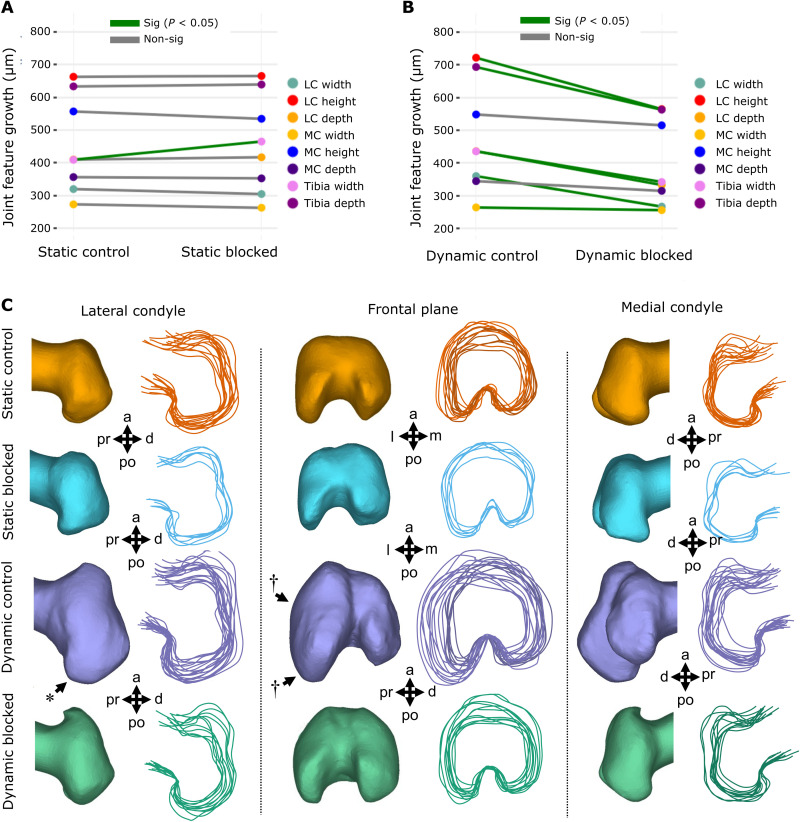
TRPV4 mechanotransduction is crucial for loading-induced joint cartilage growth and shape in dynamically cultured limbs. (**A**) Average paired differences in growth for joint cartilage features in static vehicle control limbs versus static cultured limbs exposed to TRPV4 antagonist (*n* = 6 limbs per group). Green lines, significant paired feature differences (*P* < 0.05); gray lines, nonsignificant feature differences. (**B**) Average paired differences in growth for joint cartilage features (each line represents one of eight features) in dynamic vehicle control limbs versus dynamic cultured limbs exposed to TRPV4 antagonist RN-1734 (*n* = 12 per group). Green lines, significant paired feature differences (*P* < 0.05); gray lines, nonsignificant feature differences. (**C**) Joint shape contours for distal femora cultured in static and dynamic conditions, exposed to TRPV4 antagonist RN-1734 (10 μM) or vehicle control only. *n* = 12 per group. * represents more prominent posterior curl observed in dynamically loaded limbs. † represents larger lateral expansion found in the lateral condyle of dynamically loaded limbs.

Administering the TRPV4 antagonist in static cultures had minimal effects on cartilage growth and shape, with only one measurement (tibial width, *P* < 0.05) showing significant differences between the control and blocked static limbs (Fig. 5A and fig. S1.2) and no identifiable differences in joint contours (Fig. 5C). In contrast, the inhibition of TRPV4 in dynamic limbs significantly suppressed the growth of six of eight cartilage joint features (*P* < 0.05, Fig. 5B and fig. S1.3), and joint shapes of the limbs resembled those of static distal femora (Fig. 5C). Specifically, the posterior curl protrusions in the dynamic blocked limbs were subtle in comparison to dynamic controls (Fig. 5C, *), and the outward expansion of the lateral condyle seen in the dynamic controls (Fig. 5C, †) was absent in the dynamic blocked group. Medial condyle height and depth were not significantly affected by exposure to the antagonist (Fig. 5B). The larger effect of loading on growth of the lateral condyle relative to the medial condyle is in line with the higher maximum principal stresses observed in the lateral, compared to the medial, condyle of our FE model. Together, blocking TRPV4 in static cultures has minimal effects on joint cartilage growth and shape, while blocking TRPV4 in dynamic limbs almost eliminates the growth-enhancing effects of mechanical loading on the joint cartilage, demonstrating the specific mechanoregulatory role of TRPV4 in limb development. With regard to diaphyseal mineralization, blocking TRPV4 in statically cultured limbs led to a significant (*P* < 0.05) decrease in tibial mineralization (fig. S2) but had no effect on femoral mineralization. In addition, blocking TRPV4 in dynamically cultured limbs did not affect femoral or tibial mineralization (fig. S2). Thus, it is likely that the decrease in tibial mineralization due to TRPV4 blocking in static cultures was an outlier result. Because of the lack of influx of osteogenic progenitors in the murine limb explant model, we conclude that our model is unsuitable for interrogating the role of TRPV4 activity in mineralization of the prenatal limb.

**Fig. 6.**
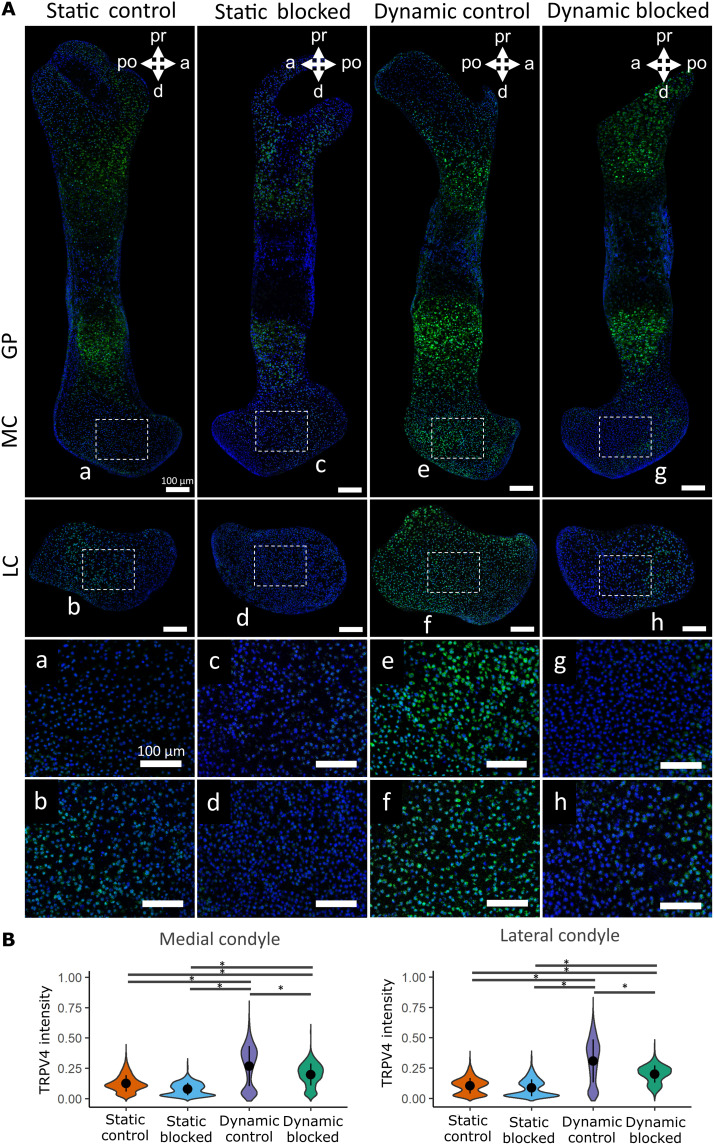
TRPV4 activity is essential for the mechanoregulation of TRPV4 expression in dynamically cultured limbs. (**A**) Immunohistochemical staining of TRPV4 protein in the medial condyle (a, c, e, and g) and lateral condyle (b, d, f, and h) of contralateral limbs subjected to static or dynamic culture conditions with the TRPV4 antagonist RN-1734 or vehicle control only (*n* = 3 limbs per group). Blue, DAPI; green, TRPV4. Boxes represent areas expanded for (**a**) to (**h**). (**B**) Quantification of pooled cell TRPV4 protein immunofluorescence intensity within the femoral condyles of static and dynamic limbs exposed to TRPV4 antagonist or vehicle control only. Black bars with * indicate a significant difference between groups (*P* < 0.05).

Statically and dynamically cultured limbs exposed to RN-1734, or the drug vehicle, were stained for the TRPV4 protein to further validate that TRPV4 expression is directly mechanoregulated and elucidate whether it is potentially self-regulated. Blocking TRPV4 activity in static cultures had no significant effects on TRPV4 regional expression (Fig. 6A) or TRPV4 expression intensity (Fig. 6B) in the condyles or in the growth plate (fig. S4). However, while TRPV4 expression in dynamic blocked condyles was still significantly higher than that seen in the static control and static blocked limbs (Fig. 6B, *P* < 0.05), expression in the dynamic blocked condyles was significantly reduced when compared to dynamic controls (Fig. 6, A and B, *P* < 0.05), indicating that TRPV4 mechanotransduction regulates TRPV4 expression in joint cartilage. In contrast, blocking TRPV4 activity in dynamic cultures had no significant effects on intensity in the growth plates (fig. S4). We conclude that TRPV4 expression and function are associated with loading-induced growth and shape in prenatal joint cartilage and that TRPV4 activity in the growth plates may be differentially regulated.

### TRPV4 mediates chondrocyte proliferation and matrix synthesis in response to loading during skeletal development

Next, we sought to determine the specific cell activities regulated by TRPV4 that drive joint development and morphogenesis. We investigated the involvement of TRPV4 activity with cell proliferation and matrix synthesis, key contributors to skeletal growth and morphogenesis (*4*, *6*). Cultured limbs were sectioned and labeled for cell mitosis with phosphohistone H3 (pHH3) using immunofluorescence techniques. The proportion of proliferating cells was calculated by normalizing to the total number of cells in the medial and lateral condyle regions. Other sections were histologically stained for sulfated glycosaminoglycans (sGAG) using Toluidine blue and for collagen using Picrosirius red. Quantification of matrix stain intensity in the joint condyle regions was used to statistically assess matrix deposition across all samples.

**Fig. 7.**
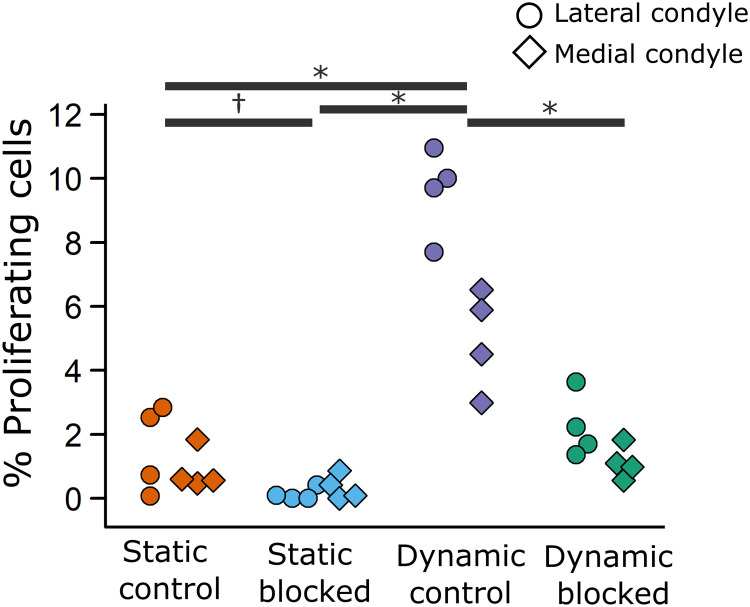
TRPV4 mechanoregulates loading-induced cell proliferation in the femoral condyles of dynamically cultured limbs. Figure illustrates the proportion of proliferating cells within the lateral and medial condyle regions, where each point represents an ensemble average of three sections from each sample (*n* = 4 limbs per group). * indicates significant difference (*P* < 0.05) between groups for the lateral and medial condyle. † indicates significant difference (*P* < 0.05) between groups for the lateral condyle only.

Subjecting the limbs to dynamic culture resulted in a significant fourfold increase in the proportion of proliferating chondrocytes in both the lateral condyle and medial condyle (Fig. 7, *P* < 0.05) compared to static culture. Notably, the proportion of proliferating cells was twofold higher in the lateral condyle compared with the medial condyle in loading conditions, in line with the greater changes in mechanically mediated growth observed in the lateral condyle compared to the medial condyle. Under static culture conditions, blocking TRPV4 led to a modest decrease in proliferation, which was only significant in the lateral condyle (Fig. 7, *P* < 0.05). Introducing the TRPV4 antagonist to dynamic limbs suppressed the mechanically induced response in both lateral and medial condyles to levels equivalent to static controls (Fig. 7, *P* < 0.05). Therefore, loading-induced proliferation in joint cartilage is strongly mediated by TRPV4 activity.

**Fig. 8.**
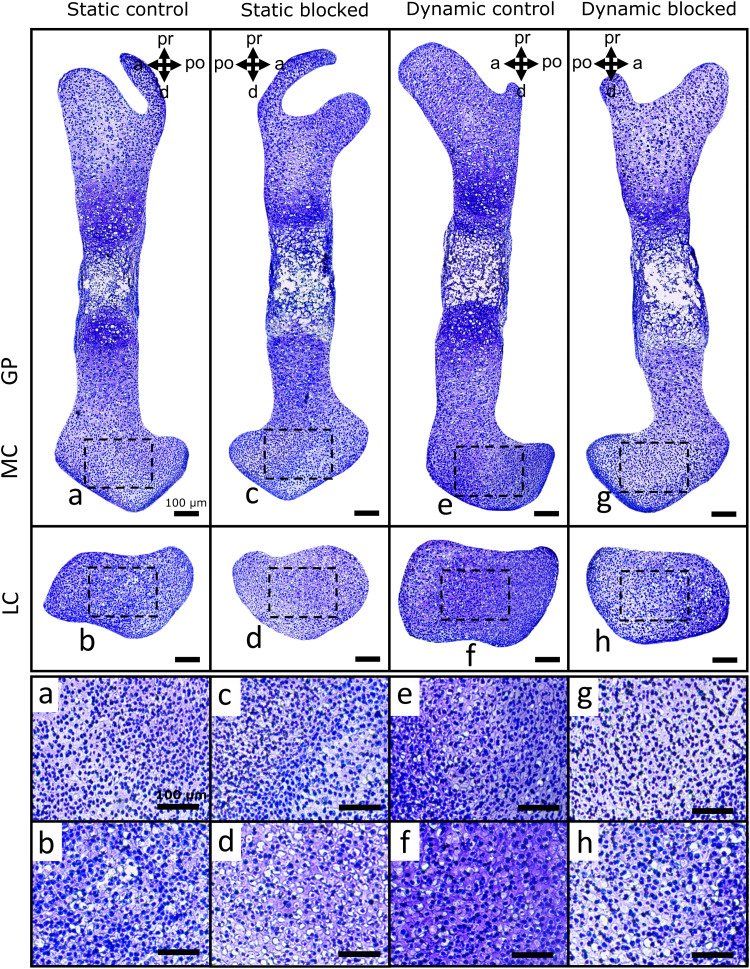
TRPV4 activity mediates loading-induced proteoglycan synthesis in the femoral condyles of dynamically cultured limbs. sGAG deposition (histologically assessed using Toluidine blue stain) in the medial condyles (**a**, **c**, **e**, and **g**) and lateral condyles (**b**, **d**, **f**, and **h**) of femora subjected to static or dynamic culture, with TRPV4 antagonist RN-1734 (10 μM) or drug vehicle (DMSO) (*n* = 3 limbs per group). Boxes represent areas expanded for (a) to (h).

**Fig. 9.**
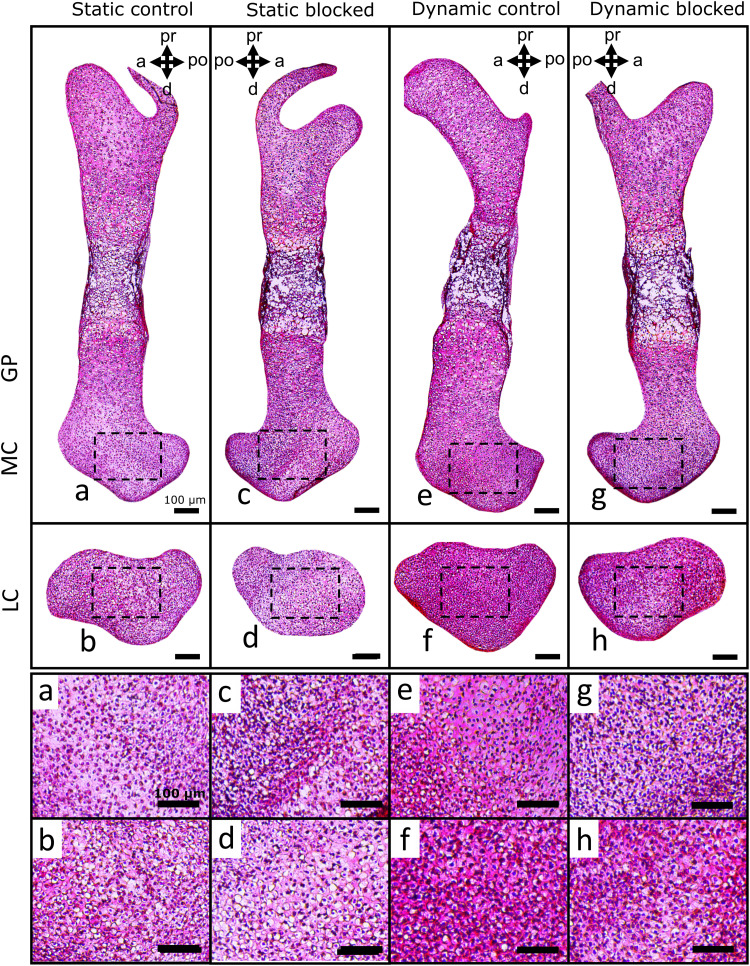
TRPV4 mechanotransduction partially regulates loading-induced collagen deposition in the femoral condyles of dynamically cultured limbs. Collagen deposition (histologically assessed using Picrosirius red) in the medial condyles (**a**, **c**, **e**, and **g**) and lateral condyles (**b**, **d**, **f**, and **h**) of femora subjected to static or dynamic culture, with TRPV4 antagonist RN-1734 (10 μM) or drug vehicle (DMSO) (*n* = 3 limbs per group). Boxes represent areas expanded for (a) to (h).

**Fig. 10.**
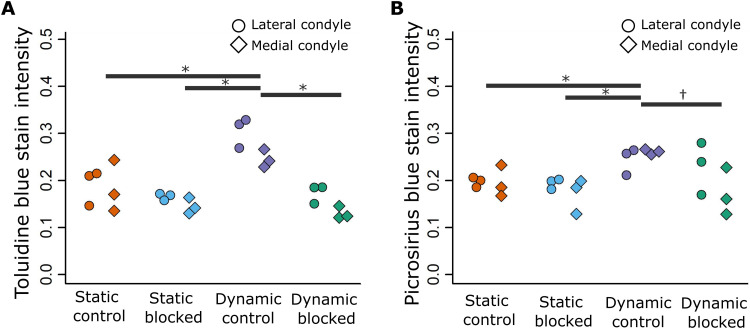
Mechanoregulated TRPV4 activity significantly mediates proteoglycan synthesis in both femoral condyles but only partially mediates collagen synthesis. (**A**) Proteoglycan deposition (histologically assessed using Toluidine blue stain) and (**B**) collagen deposition (histologically assessed using Picrosirius red) were quantified in the femoral condyles of limbs subjected to static or dynamic culture, with TRPV4 antagonist RN-1734 (10 μM) or drug vehicle (DMSO) (*n* = 3 per group). Lateral condyles, circles; medial condyles, diamonds. * indicates significant difference (*P* < 0.05) in matrix deposition in both medial and lateral condyles. † indicates significant difference (*P* < 0.05) in matrix deposition in the medial condyle only.

In terms of matrix deposition, statically cultured control limbs displayed light sGAG (Fig. 8 and fig. S5) and collagen (Fig. 9 and fig. S6) staining in the condyles, which appeared unaffected by blocking TRPV4 activity. Quantification of sGAG and collagen staining across all samples revealed no statistical differences between statically cultured limbs exposed to the vehicle or TRPV4 blocker (Fig. 10, A and B). Dynamic stimulation in control cultures potently up-regulated matrix synthesis, as both sGAG and collagen matrix were qualitatively and quantitatively increased in the condyles of dynamically cultured limbs compared to those of the static limbs (Figs. 8 to 10, *P* < 0.05). Blocking TRPV4 reduced sGAG in dynamic cultures to levels similar to those observed in static control samples (Fig. 8), while collagen deposition appeared only partially reduced in blocked dynamic cultures compared to control dynamic cultures (Fig. 9). Quantification of stain intensity corroborated the visual findings, with sGAG intensity significantly reduced in both the medial and lateral condyles of dynamically cultured limbs exposed to the TRPV4 blocker compared to dynamic vehicle control limbs (Fig. 10A, *P* < 0.05). Whereas collagen staining was only significantly reduced by TRPV4 blocking under dynamic culture in the lateral condyle (Fig. 10B, *P* < 0.05). Together, these data suggest that TRPV4 mechanotransduction in prenatal joints involves the activation of proliferation and matrix biosynthesis pathways as part of the anabolic response to dynamic loading. However, other mechanoregulatory mechanisms in addition to TRPV4 may have a substantial role in regulating collagen synthesis.

## DISCUSSION

Previous studies have identified the importance of TRPV4 in cartilage and bone mechanotransduction and, separately, the involvement of TRPV4 channelopathy in skeletal malformations, yet its role in the regulation of skeletal development has not previously been investigated. In this study, we first validated a model where we demonstrate that mechanically loading mouse embryonic limb explants ex vivo stimulates joint cartilage growth and morphogenesis but not diaphyseal mineralization. We next revealed that TRPV4 is expressed in developing cartilaginous tissues and is spatially localized to sites of high biophysical stimuli, as indicated by the correlation between regional TRPV4 expression intensity and regional stress calculated from our computational model of joint loading. We then demonstrated that TRPV4 expression is mechanically self-regulated and that regulatory pathways initiated by TRPV4 are essential for the anabolic cartilage response to physical stimuli. Last, we show that this mechanoregulatory role of TRPV4 in skeletal development is achieved via the control of cell proliferation and matrix biosynthesis. Since TRPV4 was colocalized to sites of high biophysical stimuli, we speculate that local stress patterns stimulate up-regulation of TRPV4 in the joint tissues, leading in turn to increased proliferation, matrix deposition, and consequent regional growth. This indicates a mechanism by which mechanical loading could direct morphogenesis during cartilage development and by which TRPV4 channelopathies may lead to joint malformations (*36*). Therefore, while the involvement of TRPV4 in skeletal development is evidenced by the impact of TRPV4 channelopathies on abnormal skeletal formation (*36*), this study demonstrates the specific mechanoregulatory involvement of TRPV4 in prenatal joint development.

A compelling aspect of our findings is that they reflect what have been found in cell culture studies at the cell level but, uniquely, at the cell, tissue, and organ levels in whole limb explants. Previous studies using chondrocyte-laden constructs, cultured MSCs, or chondroprogenitors found a similar dependence on TRPV4 in terms of loading-induced up-regulation of cartilage matrix gene expression markers *ACAN* and *COL2*α*1*, as well as sGAG and collagen matrix protein accumulation (*31*, *41*, *43*, *62*). Furthermore, exposing MSCs and chondrocytes to TRPV4-specific agonist GSK101 without mechanical stimulation leads to enhanced matrix synthesis at similar levels to that found in mechanically loaded cells (*31*, *43*). Together, the results from these studies combined with ours provide evidence for a critical regulatory role for TRPV4 in cartilage growth and morphogenesis. Furthermore, since our findings indicate that high TRPV4 expression localizes to regions of high mechanical stimuli, it may be possible to use mechanical stimulation combined with agonists to direct the effect of TRPV4-mediated growth to specific regions and modulate cartilage shape.

The positive effect of dynamic loading on chondrocyte proliferation in the femoral condyles of cultured mouse limbs was abolished by blocking TRPV4. While not previously investigated in chondrocytes, these findings are consistent with previous work that observed increased proliferation of micromass iPSC-derived chondroprogenitors when treated with the TRPV4 agonist GSK101 (*43*). Blocking ion channel–dependent Ca^2+^ signaling inhibits mechanically induced parathyroid hormone–related protein (PTHrP) up-regulation in chondrocytes exposed to cyclic mechanical strain (*63*). Therefore, the PTHrP/Indian hedgehog signaling loop that regulates chondrocytic proliferation and matrix synthesis (*63*, *64*) could be mediated by TRPV4. In addition to the involvement of TRPV4 activity in skeletal matrix biosynthesis and cell proliferation as identified in the present study, TRPV4 activity has also been shown to regulate chondrogenic and osteogenic differentiation of MSCs, iPSCs and chondroprogenitors (*41*, *43*–*45*). Together, these results indicate an extensive influence of TRPV4-mediated signaling over anabolic processes during skeletal development. Controlled modulation of TRPV4 may therefore be an attractive direction for enhancing the properties of developmentally inspired tissue engineered constructs that aim to recapitulate the processes of cartilage and bone development (*65*, *66*). The functional impairment induced by TRPV4 antagonists cannot be directly compared to that of TRPV4 channelopathies. However, the effects of blocking TRPV4 on joint size and shape of limbs in the present study are similar to clinically reported osteoarthropathy and joint malformations in TRPV4 channelopathies such as familiar digital arthropathy brachydactyly and spondylo-epimetaphyseal dysplasia maroteaux pseudo-morquio type 2 (*36*, *67*). The disruption of cartilage growth, morphogenesis, and cell proliferation in our dynamically cultured limbs exposed to TRPV4 blocking could be related to the stunted limb growth, epiphyseal malformations, and dysplasia observed in clinical cases.

Mechanically mediated TRPV4 expression and collagen deposition were only partially suppressed when TRPV4 was blocked in dynamically cultured limbs. This could suggest that RN-1734 did not fully block TRPV4 activity, similarly to studies that found that a different antagonist (10 μM GSK205) did not completely inhibit TRPV4 activity (*68*, *69*). In addition, Ca^2+^ signaling initiated by other mechanosensitive channels may be independently involved in anabolic mechanoregulation during development. Supportive of this hypothesis, while blocking TRPV4 with GSK205 in chondroprogenitor cells exposed to thermo-mechanical stimulation inhibits stimuli-induced up-regulation of gene expression matrix markers (*ACAN* and *COL2*α*1*), full chelation of extracellular Ca^2+^ more strongly reduces the response to stimuli (*62*), possibly indicating the involvement of Ca^2+^ channels other than TRPV4. In particular, for collagen synthesis, it is also possible that only some collagen types are regulated by TRPV4, since the Picrosirius red stain used in the present study is not selective for collagen type. Examination of the influence of mechanotransduction pathways other than TPRV4 or immunofluorescence imaging of specific collagen networks, which emerge during cartilage development (*70*), may provide further insight into the influence of different mechanosensitive Ca^2+^ channels on collagen synthesis and organization.

Other mechanosensitive ion channels such as the Piezo1 and Piezo2 channels and voltage-gated calcium channels (VGCCs) influence anabolic chondrocyte activities in response to mechanical loading. While TRPV4 channels function as transducers to physiological ranges of mechanical strain, Piezo1 and Piezo2 activation is sensitive to high-strain mechanical stress, such as hyper-physiologic levels of cell deformation (*71*, *72*). Furthermore, while both TRPV4 and Piezo1 channels contribute to currents activated by stimuli applied at cell-substrate contacts, only Piezo1 mediates stretch-activated currents (*30*). Therefore, the combination of the TRPV4, Piezo1, and Piezo2 channels gives chondrocytes the ability to respond to a continuum of mechanical strains and multimodal physical cues in cartilage. This ability may be important during development, since developing tissues undergo rapid changes in mechanical properties with growth and are exposed to varying types of mechanical input. A differential response to varying stress may lead to finer mechanoadaptive morphogenic control of the developing skeleton. Previous work from our group showed that VGCC activity is critical to mechanically induced prenatal joint cartilage morphogenesis in chick hindlimb explants (*73*), but the functional role of VGCCs in chondrocytes needs further investigation. Another study found that Ca^2+^ influx in response to hyperphysiological mechanical strain depends on the activity of L-type voltage-gated Ca^2+^ channels, indicating a regulatory signaling link between Piezo channels and VGCCs (*71*). Further investigation into the specific cell behaviors governed by various channel types may lead to a better understanding of mechanoadaptation during development and the potential for finer tuning of developmentally inspired tissue-engineered construct properties.

A limitation of the current study is that our culture model was not suitable for investigation into the involvement of TRPV4 in diaphyseal mineralization. Long bone ossification is influenced by mechanical stimulation from embryonic muscle contractions, consistently in the chick (*74*) but only in some rudiments in the mouse (*9*). Dynamic culture did not affect mineralization in the murine limb explants as it has previously in the chick hindlimb explant model for flexion-extension loading (*55*) or hydrostatic pressure (*58*). We believe that the difference between the chick and mouse limb explant studies in terms of effects of mechanical loading on ossification is because ossification in the bird long bones is intramembranous and therefore progresses in the absence of blood vessels, while endochondral ossification in mammalian long bones relies on blood vessel invasion (*3*, *59*), which does not occur in our in vitro culture system. Previous groups have developed tissue constructs mimicking aspects of endochondral ossification (*65*, *66*), which may be a more viable route to investigating the role of TRPV4 in bone development in vitro. Hydrostatic loading in vitro (*58*) could have been an alternative model to investigate mechanoregulation in our murine model. However, the effects of flexion-extension loading on collagen synthesis in the murine joints of the present study were similar to those found using hydrostatic loading in embryonic chick limb explants (*58*). Comparable findings for the joint between flexion-extension and hydrostatic pressure are expected, as compressive loading of skeletal tissues causes fluctuations in interstitial fluid pressure and thus the magnitudes of hydrostatic forces that cells in the tissues experience. While TRPV4 expression was regulated by mechanical loading in the joint cartilage, expression in the hypertrophic cartilage appeared to be consistently high and unaffected by mechanical loading. Hypertrophic chondrocytes swell to a larger cell volume when transitioning from resting chondrocytes (*3*), proposed to be a critical determinant of rudiment lengthening (*75*). We speculate that increased cell membrane tension due to hypertrophy may be intrinsically up-regulating TRPV4 expression.

While the findings from this study demonstrate that TRPV4 is a mechanoregulator of skeletal development, another limitation of this work is that chemical activation of TRPV4 in the cultured limbs was not explored as an additional positive control. Exposing MSCs and chondrocytes to the TRPV4-specific agonist GSK101 periodically for short windows can stimulate TRPV4-dependent anabolic cell activity without external mechanical stimuli (*31*, *41*, *43*). However, exposing the cultured limbs to a TRPV4 agonist would have activated the channel throughout the whole limb tissues, and our initial results demonstrated that TRPV4 needs to be activated in a site-specific manner by regional stress variations to drive joint morphogenesis. In addition, prolonged TRPV4 activation with GSK101 leads to channel desensitization and impaired chondrocyte mechanosensitivity due to loss of intracellular Ca^2+^ regulation (*76*, *77*). Therefore, it is possible that exposure of the limb explants to agonists for the long loading durations of our experiments may have been counteractive to loading-induced activation. Considerable off-target effects due to RN-1734 seem unlikely within the present study, due to the absence of effects on cartilage growth and morphogenesis or matrix deposition in statically cultured limbs. Furthermore, a study that cultured MSCs in vitro exposed to 10 μM RN-1734 continuously for 14 days did not report cell cytotoxicity (*60*). Last, while there was a clear effect of blocking TRPV4 on epiphyseal cartilage morphogenesis and matrix synthesis in dynamic cultures, we were unable to distinguish between changes in transient cartilage and future permanent articular cartilage during the stage of development assessed. Recent work from our group on characterizing collagen development in mouse embryo limbs identified no clear distinction between permanent and transient cartilage at TS25, the stage used in this study (*70*). By TS27, what we believe to be the future articular cartilage is demarcated by differential specific collagen deposition. Therefore, future investigations of later stages of joint development may shed light on the involvement of TRPV4 in articular cartilage growth and matrix maturity.

In conclusion, the results from this study indicate that TRPV4-mediated mechanotransduction is crucial for the mechanical regulation of cartilage development and morphogenesis during skeletogenesis, through, at least in part, the up-regulation of anabolic cell proliferation and matrix synthesis pathways. As a potent regulator of cartilage formation, TRPV4 may be a valuable target for the development of therapeutic disease modifying drugs or developmentally inspired tissue engineering strategies for skeletal tissue repair.

## MATERIALS AND METHODS

### Experimental design

The study was carried out with two main objectives. The first objective was to establish the effect of mechanically loading mouse embryo hindlimbs in our ex vivo bioreactor culture set up on knee joint cartilage growth and morphogenesis, diaphyseal mineralization, and local tissue expression of the TRPV4 protein in the cultured femora. For this, we compared contralateral mouse embryo hindlimbs either cultured in static conditions (static culture) or exposed to mechanical stimulation (dynamic culture). The second objective was to determine whether the first objective measures (cartilage growth, morphogenesis, and regional TRPV4 expression) affected by mechanical loading, in addition to measures representing cell anabolic behavior (cell proliferation and matrix biosynthesis), are disrupted by blocking TRPV4 with a TRPV4-specific antagonist (RN-1734). For this, we introduced static and dynamic cultured limbs with either the drug vehicle (DMSO) or the TRPV4-specific blocker (10 μM RN-1734). Statically cultured limbs exposed to the drug vehicle were compared to those cultured with the TRPV4 blocker to determine the function of TRPV4 in non–loading-dependent cell activity and identify any cytotoxic effects of the drug. Dynamically cultured limbs exposed to the drug vehicle were compared to limbs cultured with the TRPV4 blocker to reveal the involvement of TRPV4 in mechanically driven skeletal development. In summary, cultured left and right hindlimbs served as contralateral pairs for three paired comparison culture groups: (i) static versus dynamic culture, (ii) dynamic culture with DMSO versus dynamic with RN-1734, or (iii) static culture with DMSO versus static with RN-1734 (Fig. 1B).

### Limb culture

All experiments were performed in accordance with European legislation (Directive 2010/63/EU). Mouse (strain C57/B16) embryos were harvested at embryonic day 15.5 (Theiler stage 24). Hindlimbs were dissected from the spine and the bulk of soft tissue removed under the microscope to improve culture media and drug penetration. Limbs were pinned to foam supports through the superior aspect of the pelvis (Fig. 1A) and cultured in vitro at an air-liquid interface with either dynamic loading within a mechanostimulation bioreactor (movie S1) or in static conditions within a petri dish (*54*). The total culture time for both statically and dynamically cultured limbs was 6 days. Previous and preliminary work from our group determined that 6 days of culture was sufficient to produce a measurable and consistent physiological response (cartilage joint growth and morphogenesis) in mouse and chick limbs (*54*, *78*).

All culture vessels were filled with 30 ml of osteogenic media consisting of basal media (αMEM with GlutaMAX supplement), supplemented with 1% penicillian/streptomycin and amphotericin B, 100 μM ascorbic acid, 2 mM β-glycerophosphate, and 100 nM dexamethasone. TRPV4-mediated Ca^2+^ activity was blocked using RN-1734 (*60*, *61*) dissolved in DMSO (Sigma-Aldrich), diluted into the complete basal media at a final concentration of 10 μM. The concentration of RN-1734 used (10 μM) was based on a previous study exposing MSCs to the drug continuously for 14 days with no reported cytotoxic effects or cell death (*60*). The same volume of DMSO was diluted in basal media for the vehicle control group.

### Mechanical stimulation

For the dynamic culture condition, the bioreactor was programmed to apply 2-mm sinusoidal compressive displacements to the foam supports with limbs at 0.67 Hz, which induced a sagittal knee flexion of approximately 14° (±2°) (Fig. 1A and movie S1). The degree of knee flexion applied was found in previous work of our group to promote joint cartilage growth, morphogenesis, and proliferation in fetal chick limb explants (*54*, *55*, *73*). Preliminary studies (*78*) determined that the same degree of knee flexion was sufficient to generate a measurable and repeatable physiological response (cartilage growth and morphogenesis) in cultured murine embryonic limbs. Within each 24-hour period, three 2-hour intervals of mechanical stimulation were applied with 6-hour periods of rest in between. For the static culture conditions, the foam supports with limbs were placed in an enclosed petri dish.

### Cartilage and mineral measurements and shape analysis

To quantify cartilage growth and mineral length, measurements were taken from 3D representations of the hindlimb knee joints generated through OPT techniques optimized for embryo imaging (*79*). Before OPT, samples were dehydrated in ethanol, stained with 0.055% Alcian blue for 5 hours, cleared in 1% KOH for 2 hours, and then stained with 0.01% Alizarin red for 2 hours at room temperature, permitting selective visualization of the cartilaginous tissues and mineral, respectively. Following OPT scans obtained as previously described (*79*), image projections were reconstructed (NRecon, Micro Photonics Inc., USA) and segmented into 3D models (Mimics 19, Materialise, Belgium) of the cartilage and mineral. Eight knee joint cartilage features and two mineral lengths of the 3D models were measured (Fig. 2). For joint feature measurements, the distance between the apexes of anatomical landmarks was collected for each model (3-Matic, Materialise, Belgium). To assess joint feature shape, the cartilage joint models were rigidly registered using *N*-point registration (3-Matic, Materialise) and the contour of the joints extracted in the medial, frontal, and lateral views of the joints. Wilcoxon signed-rank tests with Bonferroni corrections were used to test for paired differences in quantitative cartilage growth variables and mineral length between contralateral limb samples.

### Histology and immunofluorescence microscopy

Cultured hindlimbs were processed in a sucrose gradient (15% and then 30%) and embedded in a 30% sucrose and optimal cutting temperature compound 50:50 mix. Ten-micrometer sections of the medial condyles with full-length femora and lateral condyles were collected along the sagittal axis of the femora and fixed in 4% paraformaldehyde.

For histology, frozen sections were stained with Toluidine blue (sGAG stain) for 4 min and then washed with deionized water or stained in Picrosirius red (collagen stain) for 30 min, followed by 10 min in acidified water. Sections were imaged using light microscopy with a consistent exposure time (Yenway EX30l; Life Sciences Microscope, Glasgow, UK). For immunofluorescence, frozen sections were washed with PBTD (0.1% Tween 20 and 1% DMSO in phosphate-buffered saline) for permeabilization, blocked with 5% normal goat serum for 2 hours, incubated with primary antibodies against TRPV4 protein (1:500; ab39260, Abcam) or pHH3 (1:500; ab5176, Abcam) at 4°C overnight, followed by Alexa Fluor 488–conjugated secondary antibody (1:200; ab150077, Abcam) for 2 hours and lastly stained with 4′,6-diamidino-2-phenylindole (DAPI) for 3 min.

### Quantification of TRPV4 intensity, cell proliferation, and matrix biosynthesis

To quantify average regional TRPV4 intensity, images of the growth plate and central regions of the medial condyle and lateral condyle were captured at ×40 magnification, using sections stained for TRPV4. Edges of the rudiments were not used in the quantification due to high errors in cell segmentation where the density of cell nuclei is highest. Cell nuclei were segmented as primary objects using the DAPI channel to detect chondrocyte locations, and whole-cell areas were segmented as secondary objects using the TRPV4 channel, based on the location of the primary objects (CellProfiler, Massachusetts, USA). Last, the average pixel grayscale intensity of Alexa Fluor 488 (TRPV4) was quantified within the secondary object areas. Significant differences in TRPV4 intensity (pooled across all cells) between culture groups were assessed using the Mann-Whitney *U* tests for two-group comparisons or one-way analysis of variance (ANOVA) with Tukey post hoc tests for four-group comparisons.

To quantify the percentage of proliferating cells, images taken at ×10 magnification were cropped to the medial and lateral condyle regions only, and then cell nuclei areas were segmented using the DAPI stain (CellProfiler, Massachusetts, USA) and quantified. The number of cells positively expressing pHH3 was manually counted (ImageJ, Maryland, USA).

Semiquantitative analysis of sGAG and collagen biosynthesis was achieved by measuring Toluidine blue and Picrosirius red staining intensity from histological sections. To permit comparison across samples, image acquisition was carried out using a fixed exposure time and microscope light settings across all samples. Images were verified for no over- or undersaturated pixels and a comparison of background intensity ranges of nonstained regions across all sections confirmed consistency of acquisition exposure. Ten times magnification images of stained sections were cropped to the medial and lateral condyle regions only, and nuclei were removed using a semiautomatic threshold (ImageJ, Maryland, USA). Signal intensities of the cropped images with removed nuclei were then measured to estimate the relative density of extracellular matrix (ImageJ, Maryland, USA).

### FE analysis

Principal stress was calculated through FE analysis of an ideal limb (ABAQUS, Dassault Systemes, France). Multiple 3D models generated from OPT scans of limbs were collected and separated into distinct components, namely, the distal femur and the bone collar. These components were registered using *N*-point registration, which allowed for average components to be produced. An ideal limb was created using the averaged distal femur and bone collar, with the proximal femur and tibia supplied by the clearest, most representative whole limb OPT scan (Fig. 4A). The relative orientation of the femur and tibia was adjusted to match the knee angle of limbs on the foam in the zero position from an image taken at day 6 of culture. A solid spherical joint capsule was then placed surrounding the joint space (Fig. 4A). Two boundary conditions were applied to the system. The proximal femur was driven with a ramped displacement designed to match the frequency and amplitude applied to the limbs during in vitro mechanical stimulation. The distal tibia was fixed but allowed to freely rotate, reflecting the interaction of the foot with the foam step. All components were provided with linear isotropic elastic properties, with a Young’s modulus (MPa) of 11.1 (*80*), 117 (*81*), and 0.55 (*82*) and a Poisson’s ratio of 0.49 (*80*), 0.3 (*81*), and 0.25 (*82*), for the cartilage, mineralized region, and capsule, respectively. To quantify the relationship between TRPV4 expression and stress, TRPV4 expression intensity (for the closest matching sample shape to the ideal limb) and maximum principal stresses across the distal femoral medial and lateral condyles were averaged into discrete 100-μm subregions in a grid (ImageJ, Maryland, USA). Average values from matching subregions of the medial and lateral condyles were correlated and evaluated using Pearson’s correlation with 95% confidence bands (Fig. 4C).

### Statistical analysis

Statistical tests used for each individual method are described in the relevant sections. All quantitative and qualitative assays had at least three replicates. Where only one comparison group was present, Mann-Whitney *U* tests were carried out to test for statistical significance (*P* < 0.05), while for multiple groups, ANOVA followed by Tukey post hoc with Bonferroni correction was performed to test for significance (*P* < 0.05).

## Notes

View/request a protocol for this paper from *Bio-protocol*.
