# Supplementary material for: Mechanoregulatory role of TRPV4 in prenatal skeletal development
Source: Sci Adv. 2023 Jan 25;9(4):eade2155. doi: 10.1126/sciadv.ade2155 (PMC9876556; doi:10.1126/sciadv.ade2155)
Supplement: Supplementary file 1 — Figs. S1 to S6 [file sciadv.ade2155_sm.pdf]

Science Advances
AAAS

Supplementary Materials for

Mechanoregulatory role of TRPV4 in prenatal skeletal development

Nidal S. Khatib *et al.*

Corresponding author: Niamh C. Nowlan, niamh.nowlan@ucd.ie

Sci. Adv. **9**, eade2155 (2023)
DOI: 10.1126/sciadv.ade2155

The PDF file includes:

Figs. S1 to S6
Legend for movie S1

Other Supplementary Material for this manuscript includes the following:

Movie S1

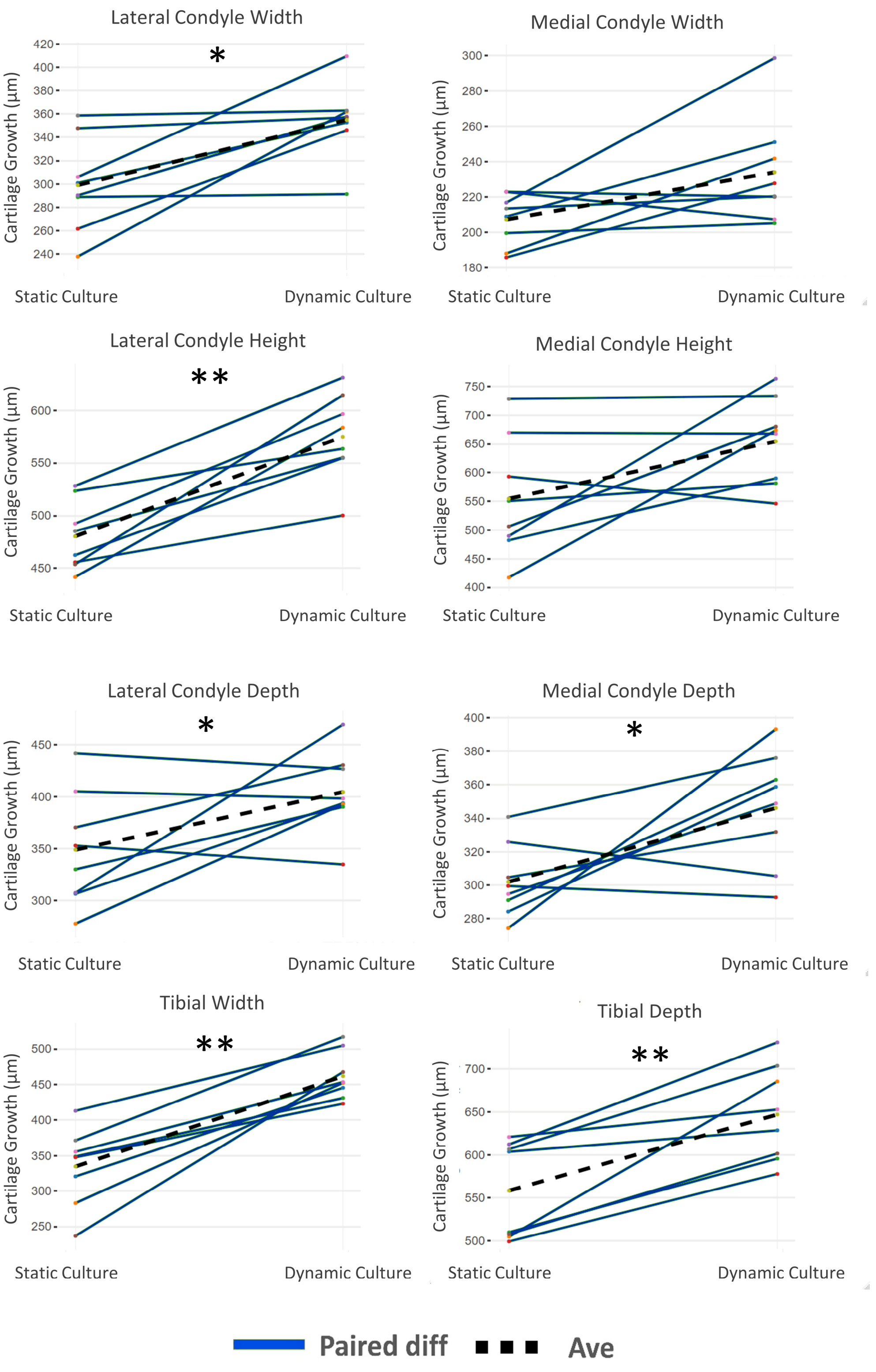

Fig. S1.1. Paired sample differences in joint cartilage feature growth between statically and dynamically cultured limbs. Each line shows the difference between contralateral limbs of one embryo exposed to either static culture (petri dish) or dynamic culture (mechanical stimulation). * $p<0.05$; ** $p<0.01$; n=8 limbs per condition.

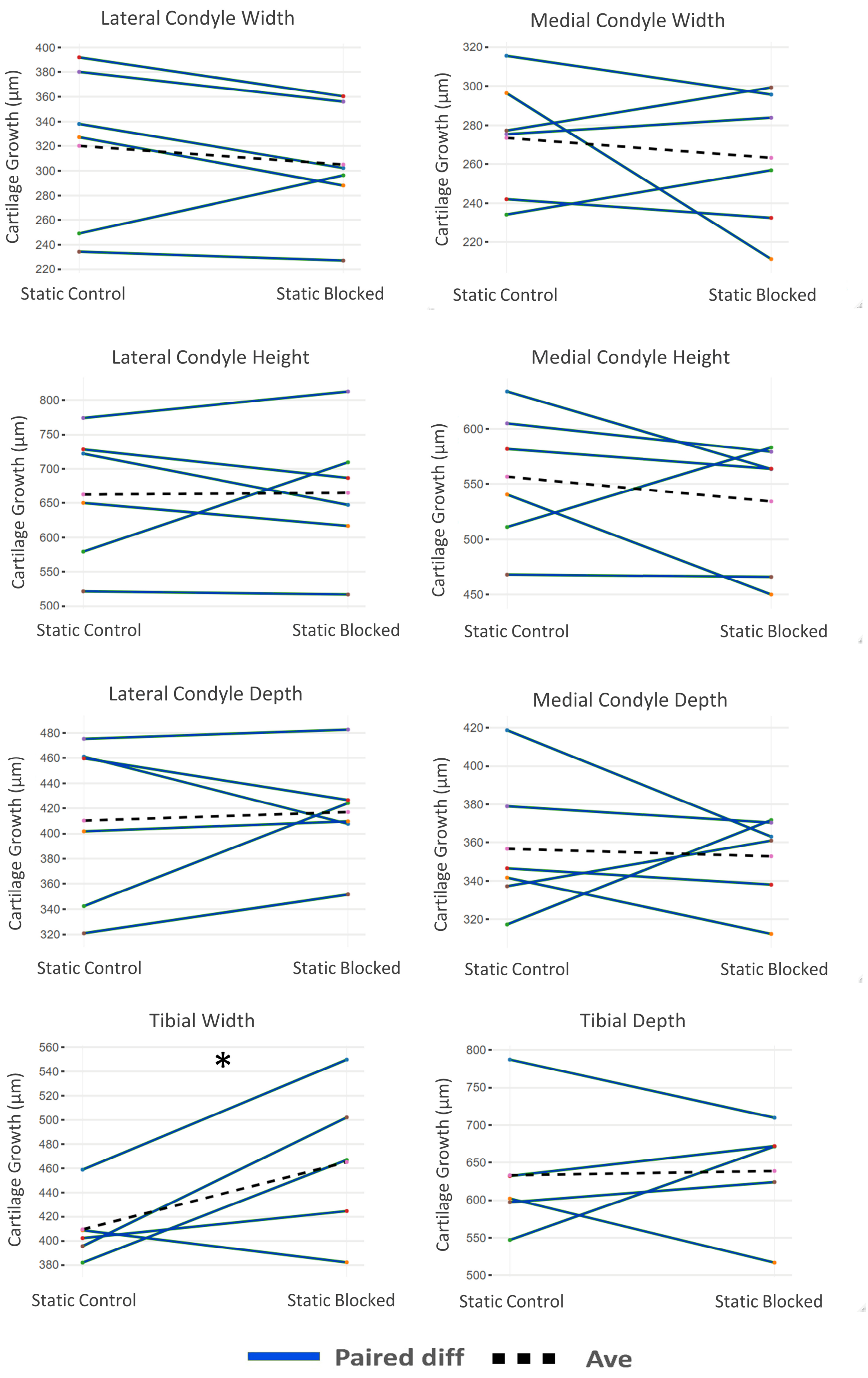

Fig. S1.2. Paired sample differences in joint cartilage feature growth between static vehicle control and static blocked limbs. Each line shows the difference between contralateral limbs of one embryo exposed either static culture with the blocker (10µM RN-1734) or with the drug vehicle only (DMSO). * $p<0.05$; ** $p<0.01$; n=5-6 limbs per condition.

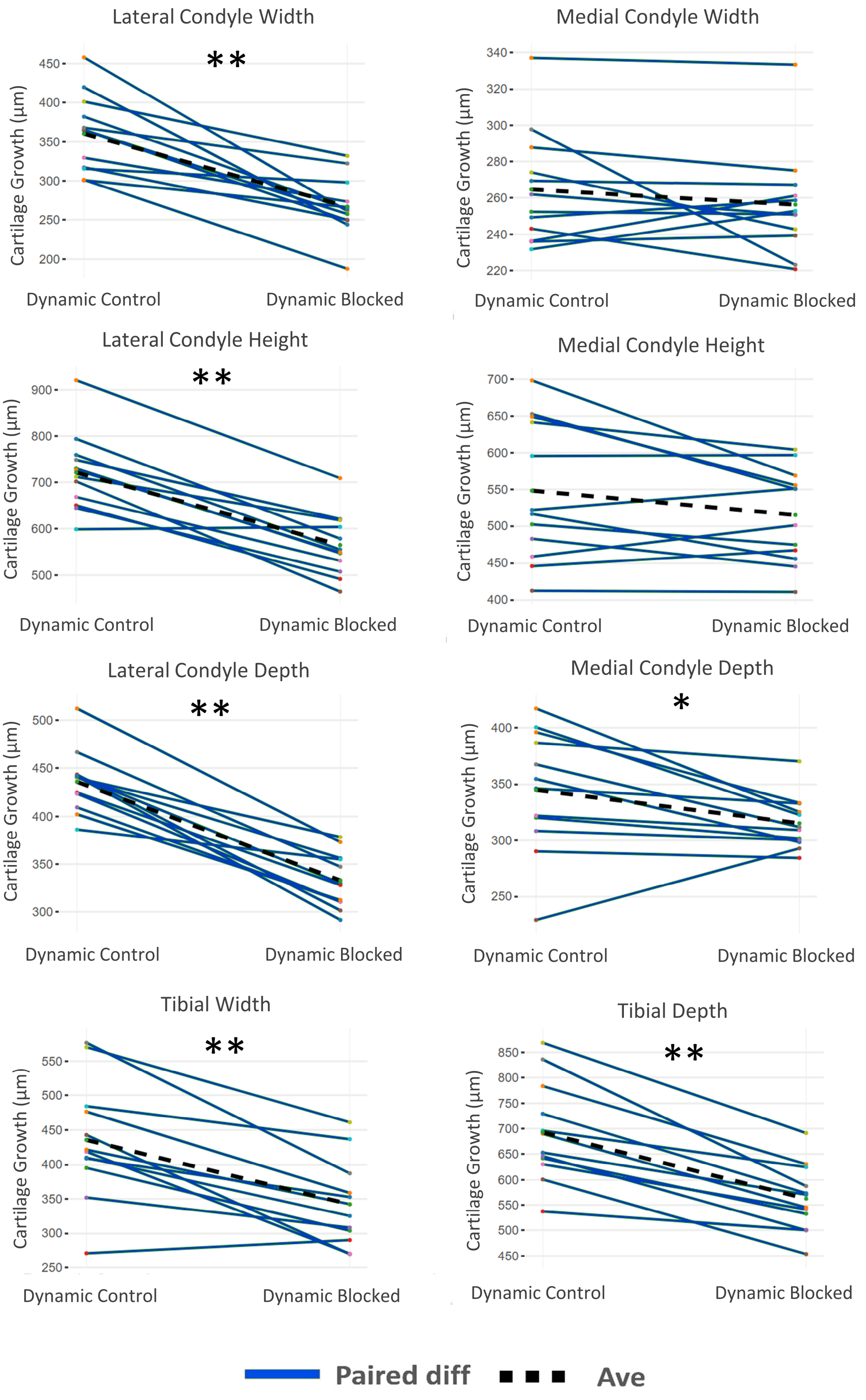

Fig. S1.3. Paired sample differences in joint cartilage feature growth between dynamic control and dynamic blocked limbs. Each line shows the difference between contralateral limbs of one embryo exposed to either dynamic culture with the blocker (10µM RN-1734) or with the drug vehicle only (DMSO). * p<0.05; ** p<0.01; n=12 limbs per condition.

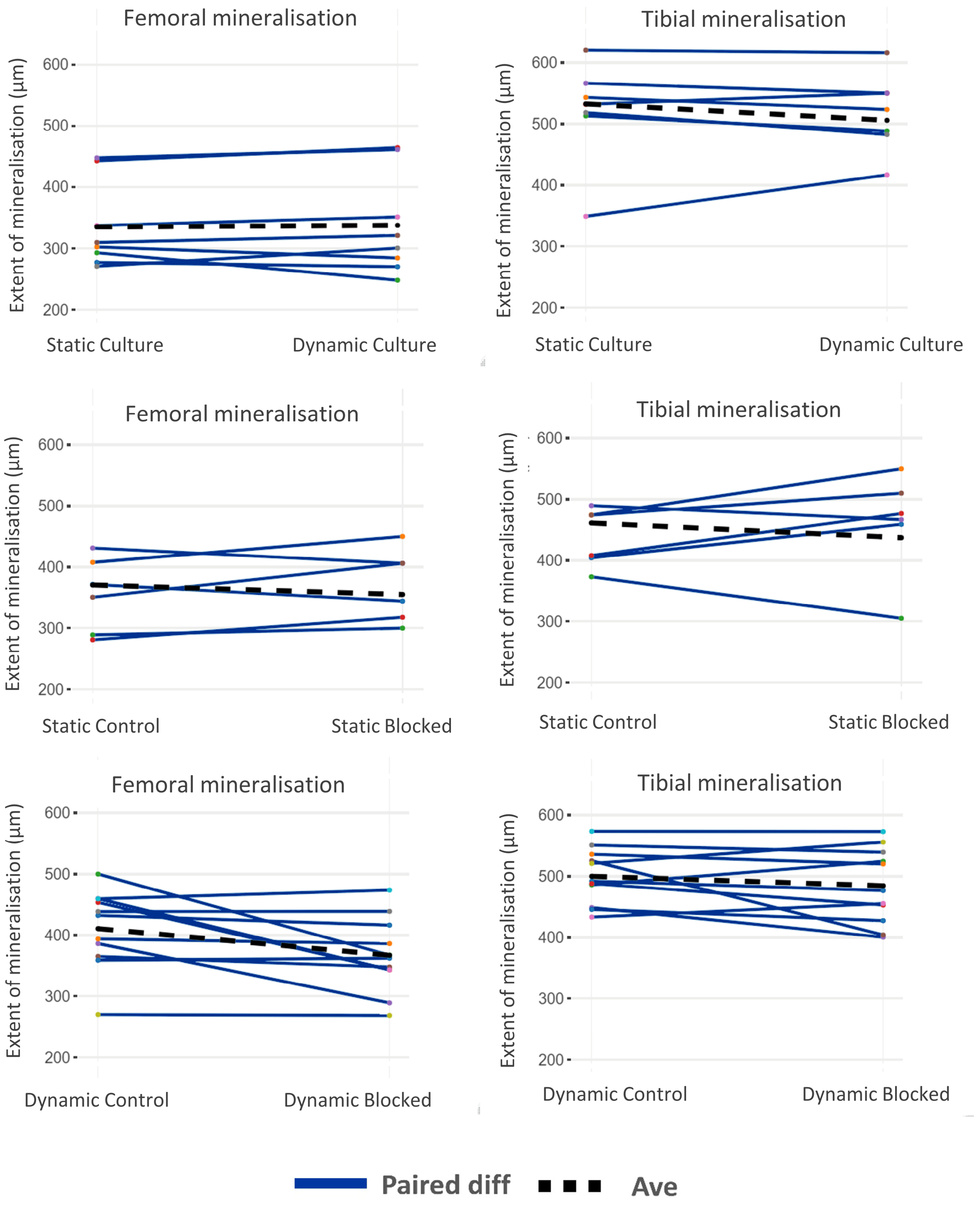

Fig S2. Paired sample differences in diaphyseal mineralization in all comparison groups. Each line shows the difference between contralateral limbs of one embryo. * $p<0.05$; ** $p<0.01$; static vs dynamic, n=6 limbs per condition; dynamic control vs dynamic blocked, n=10 limbs per condition, dynamic control vs dynamic blocked, n=6 limbs per condition.

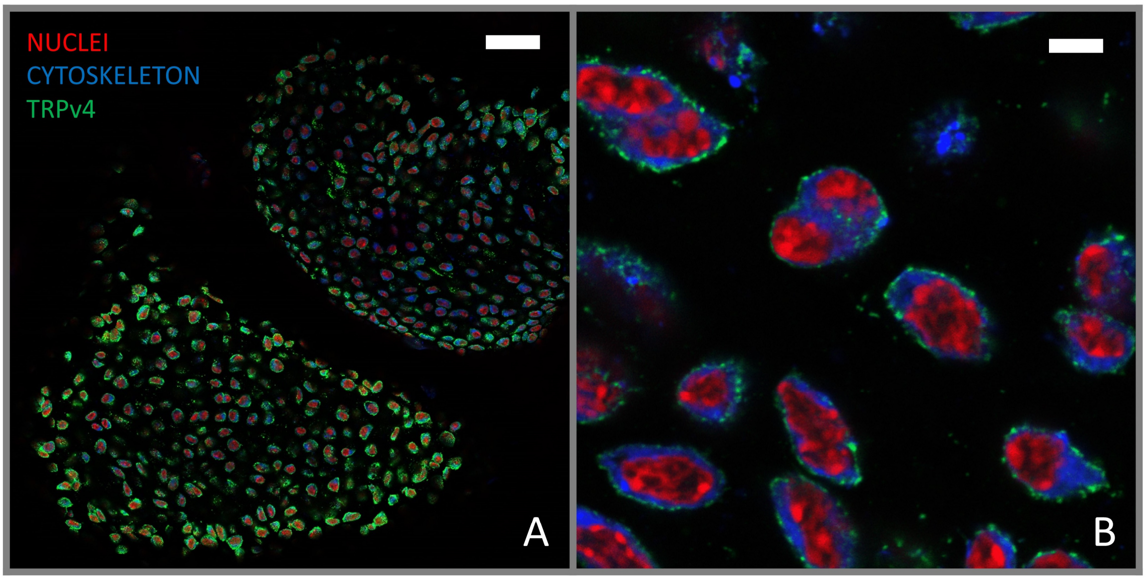

Fig. S3. Immunolocalization of TRPv4 on the cell membrane of embryonic murine epiphyseal chondrocytes. Red; nuclei, blue; cell cytoskeleton, green; TRPv4 immunolocalization. Scale bars: 50µm for A and 10µm for B.

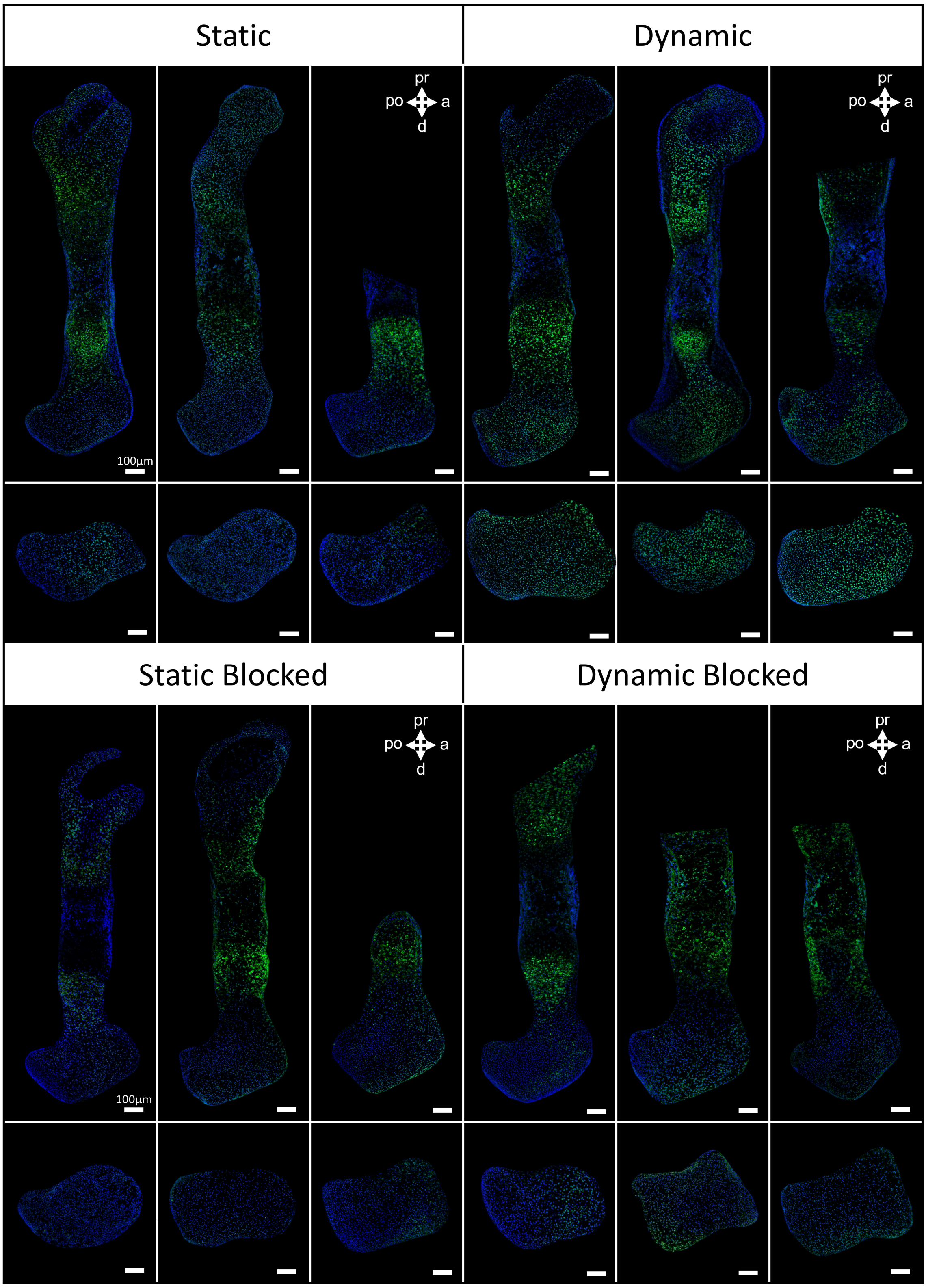

Fig S4. Regional TRPV4 protein expression within all mouse embryo femora. Each column represents contralateral limbs from a single embryo split into two comparison groups. The static and dynamic vehicle control limbs (top row) are horizontally mirrored to aid comparison across the whole dataset. n=3 limbs per comparison group.

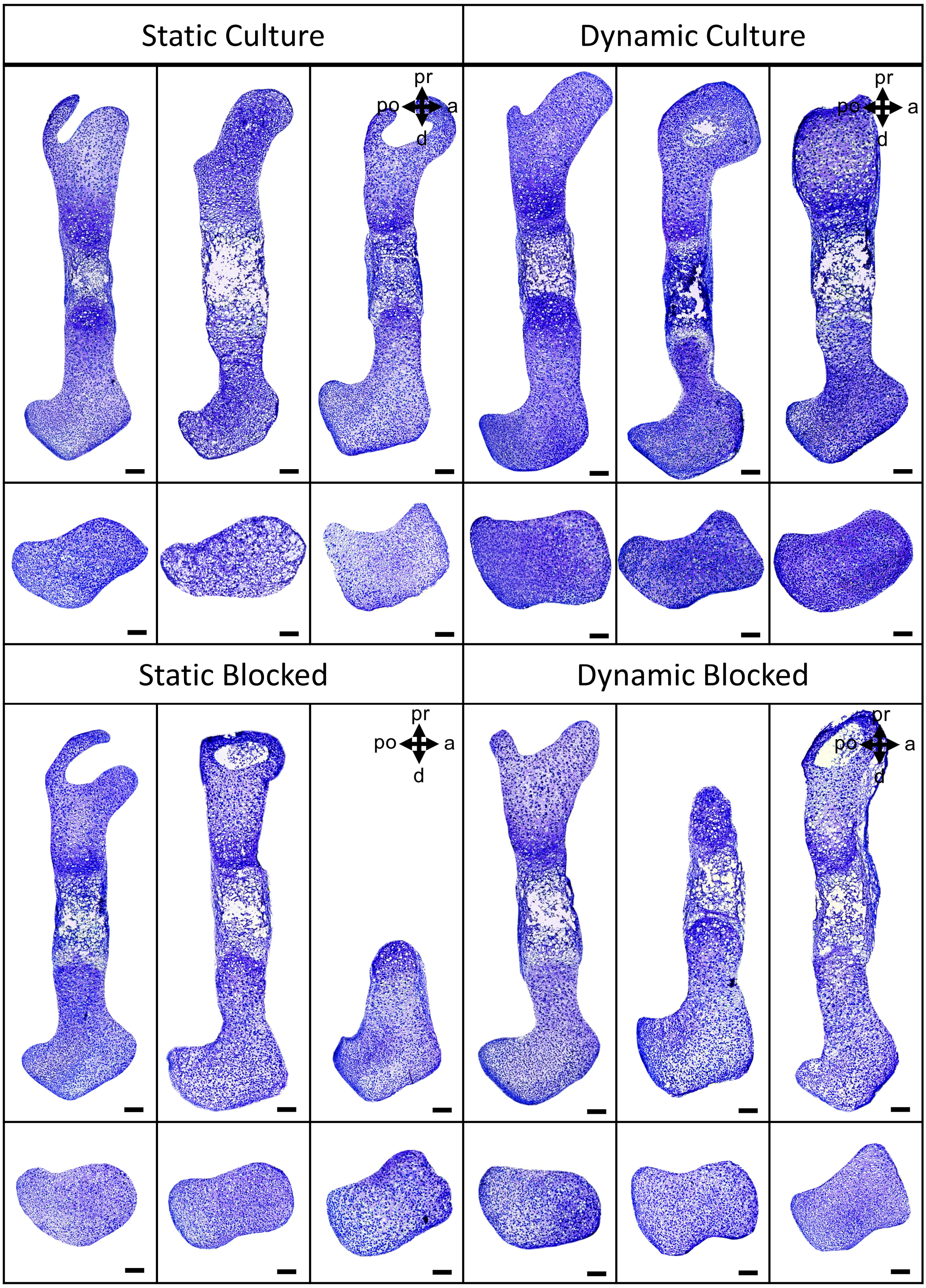

Fig. S5. Glycosaminoglycans localization within all mouse embryo femora. Each column represents contralateral limbs from a single embryo in two comparison groups. The static and dynamic vehicle control limbs (top row) are horizontally mirrored to aid comparison across the whole dataset. n=3 limbs per comparison group.

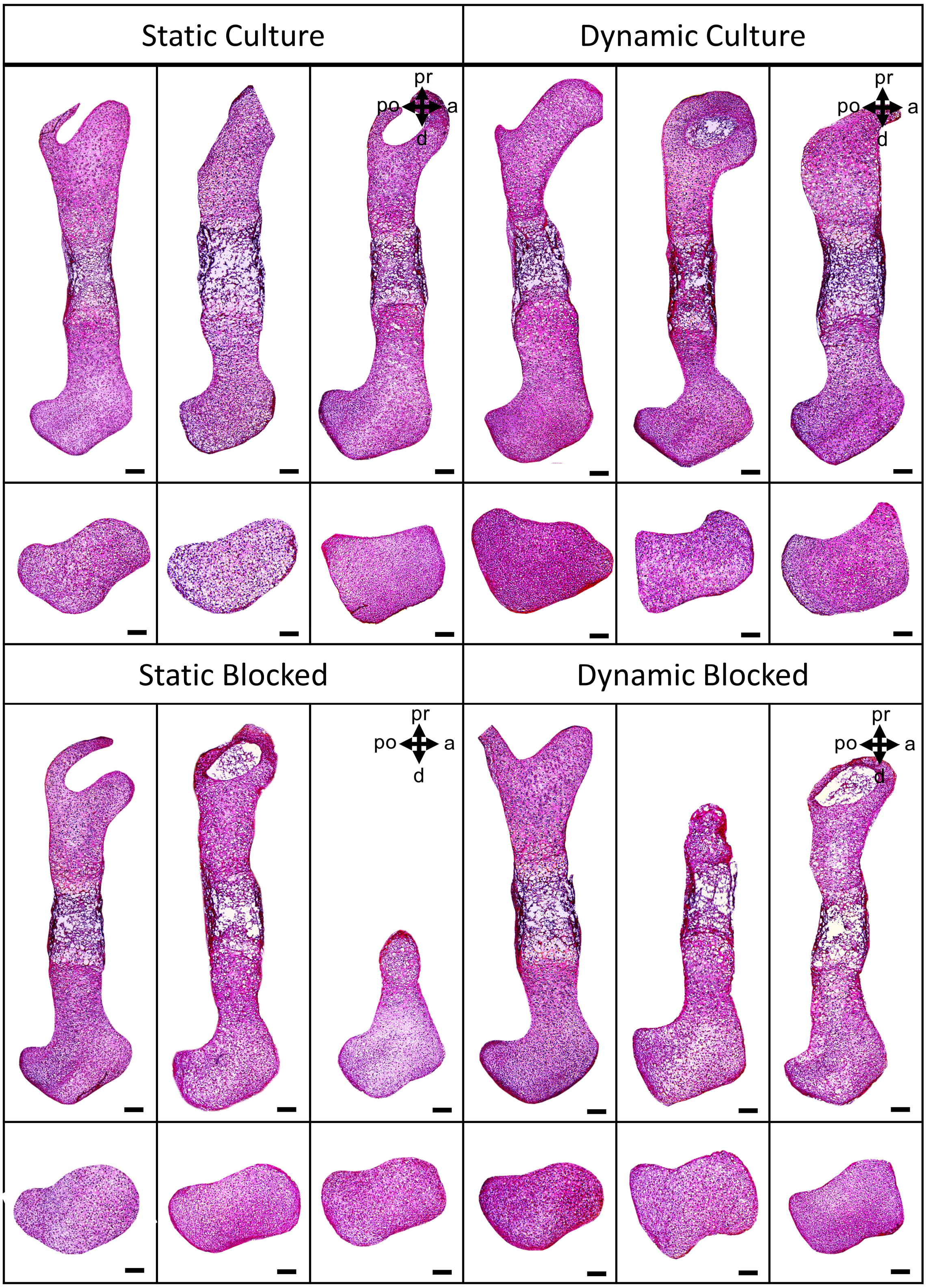

Fig. S6. Collagen localization within all mouse embryo femora. Each column represents contralateral limbs from a single embryo in two comparison groups. The static and dynamic vehicle control limbs (top row) are horizontally mirrored to aid comparison across the whole dataset. n=3 limbs per comparison group.

Movie S1. Movie illustrating the mechanostimulation bioreactor ex vivo hindlimb culture set-up during a mouse embryo hindlimb loading period. Six to eight limbs pinned to foam supports were placed within bioreactor chambers for dynamic culture or petri dishes for static culture. Dynamic cultured limbs were exposed to cyclic flexion-extension movements of approximately 14° (±2°) at 0.67Hz, applied by compressive displacement of the foam supports.
